# Updating the Definition of the Alcohol Hangover

**DOI:** 10.3390/jcm9030823

**Published:** 2020-03-18

**Authors:** Joris C. Verster, Andrew Scholey, Aurora J. A. E. van de Loo, Sarah Benson, Ann-Kathrin Stock

**Affiliations:** 1 Division of Pharmacology, Utrecht Institute for Pharmaceutical Sciences (UIPS), Utrecht University, 3584CG Utrecht, The Netherlands; j.c.verster@uu.nl (J.C.V.);; 2 Institute for Risk Assessment Sciences (IRAS), Utrecht University, 3584CM Utrecht, The Netherlands; 3 Centre for Human Psychopharmacology, Swinburne University, Melbourne, VIC 3122, Australia; andrew@scholeylab.com (A.S.); Sarahmichellebenson@gmail.com (S.B.); 4 Cognitive Neurophysiology, Department of Child and Adolescent Psychiatry, Faculty of Medicine, TU Dresden, Fetscherstr. 74, 01307 Dresden, Germany

**Keywords:** alcohol, hangover, definition

## Abstract

In 2016, the Alcohol Hangover Research Group defined the alcohol hangover as “the combination of mental and physical symptoms experienced the day after a single episode of heavy drinking, starting when blood alcohol concentration (BAC) approaches zero”. In the light of new findings and evidence, we carefully reviewed the different components of that definition. Several studies demonstrated that alcohol hangovers are not limited to heavy drinking occasions. Instead, data from both student and non-student samples revealed that at a group level, alcohol hangover may occur at much lower BAC levels than previously thought. Regression analysis further revealed that for individual drinkers, the occurrence of hangovers is more likely when subjects consume more alcohol than they usually do. However, hangovers may also occur at a drinker’s usual BAC, and in some cases even at lower BAC (e.g. in case of illness). We also carefully reviewed and modified other parts of the definition. Finally, hangovers are not necessarily limited to the ‘next day’. They can start at any time of day or night, whenever BAC approaches zero after a single dinking occasion. This may also be on the same day as the drinking occasion (e.g. when drinking in, or until the morning and subsequently having a hangover in the afternoon or evening). To better reflect the new insights and sharpen the description of the concept, we hereby propose to update the definition of the alcohol hangover as follows: “The alcohol hangover refers to the combination of negative mental and physical symptoms which can be experienced after a single episode of alcohol consumption, starting when blood alcohol concentration (BAC) approaches zero”, and recommend to use this new definition in future hangover research.

## 1. Introduction

In 2016, the Alcohol Hangover Research Group defined the alcohol hangover as “the combination of mental and physical symptoms experienced the day after a single episode of heavy drinking, starting when blood alcohol concentration (BAC) approaches zero” [1]. The development of this definition was a welcome and necessary addition to the substance abuse and addiction research field. Since then, ongoing research has generated new insights and there have been continuous discussions among researchers about how to further improve the definition of the alcohol hangover. Updating the current definition is necessary to describe the alcohol hangover more precisely against the background of new findings in the field. These specifications address recently discussed issues and further remove ambiguity from the previous wording.

## 2. Heavy Drinking 

The most important discussion pertains to the amount of alcohol consumption that is required to elicit a hangover. Given this discussion, there is controversy about the word ‘heavy’ in the definition of alcohol hangover. First, the word ‘heavy’ is unspecific, as it does not define what exact amount of alcohol should actually be consumed to elicit a hangover. Second, it suggests that hangovers occur only when large amounts of alcohol are consumed. However, the Alcohol Hangover Research Group recently reached a consensus to abandon the criterion that a BAC of 0.11% or higher is needed to provoke a hangover [2]. This conclusion was drawn based on an increasing body of evidence showing that drinkers also report hangovers at BACs that are much lower than both the suggested threshold of 0.11%, as well as the binge drinking threshold of 0.08% issued by the National Institute on Alcohol Abuse and Alcoholism (NIAAA) [3]. 

For example, Verster et al. [4] found that non-student subjects (*N* = 176) who consumed a mean (SD) of 3.0 (1.8) alcoholic drinks (10 g ethanol each) reported considerable next-morning hangover severity, i.e., a mean (SD) overall hangover severity score of 4.6 (2.4) on a 0–10 scale. Despite this, their peak estimated BAC was 0.03%. In another student sample, Kruisselbrink et al. [5] found significant hangover symptoms in subjects having consumed as few as two beers, with a mean maximum BAC of 0.036%. These observations are neither consistent with ‘heavy drinking’ in the definition of the alcohol hangover, nor with the binge drinking threshold suggested by the NIAAA. Surveys completed by large student samples from Canada (*N* = 5540) and The Netherlands (*N* = 6002) further confirmed that alcohol hangovers are reported across all BAC levels [6,7]. Thus, hangovers may occur at any reasonable BAC level, and are not limited to ‘heavy’ drinking only. Given this, we need to modify the current definition of the alcohol hangover and omit the referral to heavy drinking.

## 3. The Concept of Alcohol Hangover Versus Risk Factors and Possible Causes

When developing a definition, it is vital to accurately describe the concept (i.e., alcohol hangover). Furthermore, a proper definition of a phenomenon should not contain potential risk factors for its occurrence. There are many risk factors for hangover including, but not limited to, the amount of consumed alcohol (compared to normal), peak BAC, congener content of drinks, smoking, activities during drinking (e.g. dancing or sitting in a bar), or the emotional state during drinking. While these risk factors are of course important to investigate and mention in relation to alcohol hangover, they should not be included in a definition of the concept itself, as the observation/diagnosis of a condition should be separate from the risk/likelihood that it will occur. It is however important to still refer to ‘alcohol consumption’ in the definition of alcohol hangover as this behavior is mandatory to elicit the condition (rather than a mere risk factor). 

Evaluating hangover experiences from individual drinkers has shown that developing a useful, short, and accurate description of the relevant amount of alcohol intake to elicit a hangover is not straightforward. First of all, the presence and severity of alcohol hangovers may vary from day to day [8], even when the same amount of alcohol is consumed and the same BAC is reached. In line with this, regression analyses revealed that neither the amount of consumed alcohol, nor BAC, were strong predictors of hangover severity [2]. Instead, the relative increase in alcohol consumption, as compared to what subjects normally consume on a typical drinking occasion, was the best predictor of overall hangover severity [2]. Thus, the chances of having a hangover are significantly increased when drinking more alcohol than usual, whatever the usual amount consumed. However, including the phrase ‘relatively elevated amounts of alcohol consumption’ into the definition would exclude a substantial amount of drinkers who also experience hangovers, but do not fulfill this criterion. For example, there are drinkers who almost always experience a hangover, also when only drinking their usual amount of alcohol. One of the many potential reasons for this could be deficient metabolization of alcohol and/or its metabolite acetaldehyde in the liver. Genetic variation in alleles for alcohol dehydrogenase (ADH) and aldehyde dehydrogenase (ALDH), which are the enzymes necessary for metabolizing ethanol into acetaldehyde and further into acetate, may account for this. Twin studies showed that heritability of this genetic variation is related to about 45% of the reported hangover severity [9,10]. In this context, in populations of Asian descent, subjects with ALDH2*2 alleles, i.e., those who breakdown acetaldehyde more slowly, usually report significantly worse hangovers [11,12], and are more likely to experience hangovers at lower alcohol consumption levels than others. In such hangover-sensitive drinkers, the amount of alcohol does not need to be elevated to elicit a hangover. Aside from this, hangovers may also occur when drinking less alcohol than usual on a given occasion. This might for example be the case if subjects experience illness or reduced immune fitness [13,14], or in case of elevated negative mood while drinking [15,16]. It is hence challenging to encompass the different scenarios that may result in a hangover in a modified definition, without making it very lengthy. We therefore propose to substitute ‘heavy drinking’ with ‘alcohol consumption’ without any further reference to the amount consumed. Further, to more accurately reflect the day-to-day variability in the likelihood of developing a hangover despite more or less equal circumstances [8], we further propose to change ‘experienced’ into ‘which can be experienced’. This also acknowledges the fact that about 10% to 20% of drinkers report not having a hangover, even after consuming large amounts of alcohol [6,7].

## 4. Alcohol Hangover Symptoms

The definition refers to a ‘combination of mental and physical symptoms’. Hangover symptoms are generally perceived to be negative, but the original wording does not specify whether these symptoms are expected to be negative or positive. Therefore, we suggest to modify this as a ‘combination of negative mental and physical symptoms’. In line with previous discussions [1], hangover symptoms are not listed as part of the definition. Symptoms vary between drinkers and between drinking occasions, even when same amounts of alcohol are consumed [8,17]. Including specific symptoms instead of the general description ‘combination of negative mental and physical symptoms’ would thus significantly limit the applicability of the definition.

## 5. Timing of Drinking

The previous definition states that hangover is ‘experienced the day after…’, which was included to clearly differentiate the intoxication phase from the hangover phase. In the vast majority of cases, the hangover starts when waking up after an afternoon, evening, or night of drinking, followed by a period of sleep. Yet, this definition would not properly match cases where an individual drinks past midnight, in the morning, or during the day [18]. For example, a UK study revealed that almost 20% of all ‘drinking occasions’ took place before 5:00 p.m. [19]. In these instances, drinkers may experience a hangover in the afternoon or evening of the same day. We therefore decided to omit the next day criterion and changed the definition to ‘experienced after’.

## 6. Differentiating between Alcohol Hangover and Withdrawal

The definition refers to ‘a single episode…’. This was included to differentiate hangovers in social drinkers from withdrawal symptoms experienced by individuals with alcohol use disorders (i.e., alcoholism), who tend to not only engage in alcohol binges but also maintain a rather steady baseline level of alcohol consumption with continuous drinking for several days, or even longer. This leads to extensive homeostatic adaptations in the regulation of many vital parameters as well as neuroadaptive processes [20], which foster the development of alcohol tolerance. These counter-regulatory mechanisms require clinical treatment as they may cause life-threatening complications when the BAC approaches zero. In contrast to individuals with alcohol use disorder, social drinkers lack such extensive tolerance, as they do by definition not engage in such continuous drinking. Given the functional differences in the symptom-associated drinking patterns as well as the underlying physiological mechanisms, we therefore decided not to alter this part of the definition.

## 7. Differentiating between Alcohol Hangover and Intoxication 

The definition states that hangover is ‘starting when blood alcohol concentration (BAC) approaches zero’. This is crucial in order to clearly distinguish between alcohol intoxication and alcohol hangover on the basis of timing. We did therefore not alter this part of the definition.

## 8. Conclusions

We hereby propose to update the definition of alcohol hangover as follows: “The alcohol hangover refers to the combination of negative mental and physical symptoms which can be experienced after a single episode of alcohol consumption, starting when blood alcohol concentration (BAC) approaches zero”. 
